# Scavenging of reactive oxygen species by phenolic compound-modified maghemite nanoparticles

**DOI:** 10.3762/bjnano.10.108

**Published:** 2019-05-20

**Authors:** Małgorzata Świętek, Yi-Chin Lu, Rafał Konefał, Liliana P Ferreira, M Margarida Cruz, Yunn-Hwa Ma, Daniel Horák

**Affiliations:** 1 Institute of Macromolecular Chemistry, Czech Academy of Sciences, Heyrovského nám. 2, Prague 6 162 06, Czech Republic; 2 Department of Physiology and Pharmacology and Healthy Aging Research Center, College of Medicine, Chang Gung University, Guishan, Taoyuan 33302, Taiwan, ROC; 3 Physics Department, University of Coimbra, Coimbra 3004-516, Portugal; 4 BioISI, Biosystems and Integrative Sciences, Faculdade de Ciências, Universidade de Lisboa, Lisboa 1749-016, Portugal

**Keywords:** antioxidants, chitosan, maghemite nanoparticles, oxidative stress, phenolic compound

## Abstract

Maghemite (γ-Fe_2_O_3_) nanoparticles obtained through co-precipitation and oxidation were coated with heparin (Hep) to yield γ-Fe_2_O_3_@Hep, and subsequently with chitosan that was modified with different phenolic compounds, including gallic acid (CS-G), hydroquinone (CS-H), and phloroglucinol (CS-P), to yield γ-Fe_2_O_3_@Hep-CS-G, γ-Fe_2_O_3_@Hep-CS-H, and γ-Fe_2_O_3_@Hep-CS-P particles, respectively. Surface modification of the particles was analyzed by transmission electron microscopy, dynamic light scattering, attenuated total reflection Fourier transform infrared spectroscopy, and thermogravimetric analysis. Magnetic measurements indicated that the polymer coating does not affect the superparamagnetic character of the iron oxide core. However, magnetic saturation decreased with increasing thickness of the polymer coating. The antioxidant properties of the nanoparticles were analyzed using a 2,2-diphenyl-1-picrylhydrazyl (DPPH) assay. Cellular uptake and intracellular antioxidant activity of the particles were evaluated by an iron assay and flow cytometry, respectively, using L-929 and LN-229 cells. Compared to the control, the phenolic modification significantly reduced intracellular reactive oxygen species (ROS) levels to 35–56%, which was associated with a 6–8-times higher cellular uptake in L-929 cells and a 21–31-times higher cellular uptake in LN-229 cells. In contrast, γ-Fe_2_O_3_@Hep particles induced a 3.8-times and 14.9-times higher cellular uptake without inducing antioxidant activity. In conclusion, the high cellular uptake and the antioxidant properties associated with the phenolic moieties in the modified particles allow for a potential application in biomedical areas.

## Introduction

Reactive oxygen species (ROS) play a critical role in maintaining homeostasis in living organisms because they participate in cell-signaling pathways that control programmed cell death, gene expression, and mechanisms of immune defense [1–2]. Excessive ROS are undesirable because they lead to oxidative stress, inflammation, aging in general, and the development of fatal diseases, such as cancer, neurodegenerative disorders, atherosclerosis, heart failure, myocardial infarction, and infections. ROS of radical origin (superoxide and hydroxyl) are particularly harmful because they initiate oxidative chain reactions that induce irreversible fatal damage (methylation) of cellular nucleic acids and the oxidation of unsaturated fatty acids of cell membranes. Living organisms have evolved with several strategies, such as repair mechanisms and physical, enzymatic and non-enzymatic antioxidant defenses, to control ROS levels and protect themselves from the deleterious impact of uncontrolled ROS activity. The enzymatic antioxidant defense involves catalase, superoxide dismutase, and glutathione peroxidase, which convert harmful oxidative products first to hydrogen peroxide and then to water. These processes are catalyzed by metal-ion cofactors, such as iron, copper, zinc, and manganese [3]. The non-enzymatic antioxidant defense uses glutathione, vitamins C and E, melatonin, catecholamines, and substances of plant origin, such as phenols and carotenoids, to interrupt undesirable ROS action. Phenolic compounds are found in tea, coffee, chocolate, cacao, thyme, berries, spinach, and many other food sources [4]. The efficacy of ROS inactivation by phenolic compounds mainly depends on their chemical structure. The simplest phenolic compound, phenol, contains only one benzene ring and one hydroxy group, whereas flavonoids and lignins have many functional groups attached to the multiple benzene rings within a single molecule. The simplest phenolic compounds consist of only one aromatic ring and a type, number, and arrangement of functional groups that strongly determine the antioxidant activity [5]. Generally, phenolic compounds with a higher number of hydroxy groups have enhanced ROS scavenging capability. In addition to low-molecular-weight phenolic compounds of natural origin, some inorganic nanoparticles also have antioxidant properties, e.g., zinc, cerium, magnesium oxide, magnetite, and silver [6–11]. To enhance the antioxidant properties of inorganic particles, they should be surface-modified with antioxidants or antioxidant-modified polymers. These types of polymers include chitosan, which is a product of chitin deacetylation and is composed of D-glucosamine and *N*-acetyl-D-glucosamine [12]. Due to its biocompatibility, it has been investigated in several biomedical applications, e.g., tissue engineering, ophthalmology, and drug delivery. Chitosan modification with phenolic compounds leads to the enhancement of already existing antioxidant properties [13]. The antioxidant properties of chitosan increase with an increasing degree of deacetylation and a decreasing molecular weight [14–15]. Three strategies for incorporating phenolic compounds into chitosan have previously been described, including laccase- or tyrosinase-mediated enzymatic grafting, carbodiimide-activated conjugation via esterification or amidation, and free-radical grafting [16].

The aim of this work was to design and fabricate superparamagnetic iron oxide nanoparticles with antioxidant properties. Positively charged γ-Fe_2_O_3_ nanoparticles were synthesized through co-precipitation, and their surface was modified with polymers via a layer-by-layer (LbL) technique [17]. Two naturally derived polymers, namely, anionic heparin and cationic chitosan, were used as nanoparticle coatings, and three phenolic compounds, including hydroquinone, phloroglucinol, and gallic acid, differing in the number of hydroxy groups on the aromatic ring, were used to modify chitosan. The antioxidant properties of neat and modified γ-Fe_2_O_3_ particles were investigated in vitro in terms of their ability to scavenge ROS and thus minimize the oxidative burst in L-929 and LN-229 cells.

## Results and Discussion

### Chitosans modified with phenolic compounds

#### Physicochemical characterization

**Figure 1:**
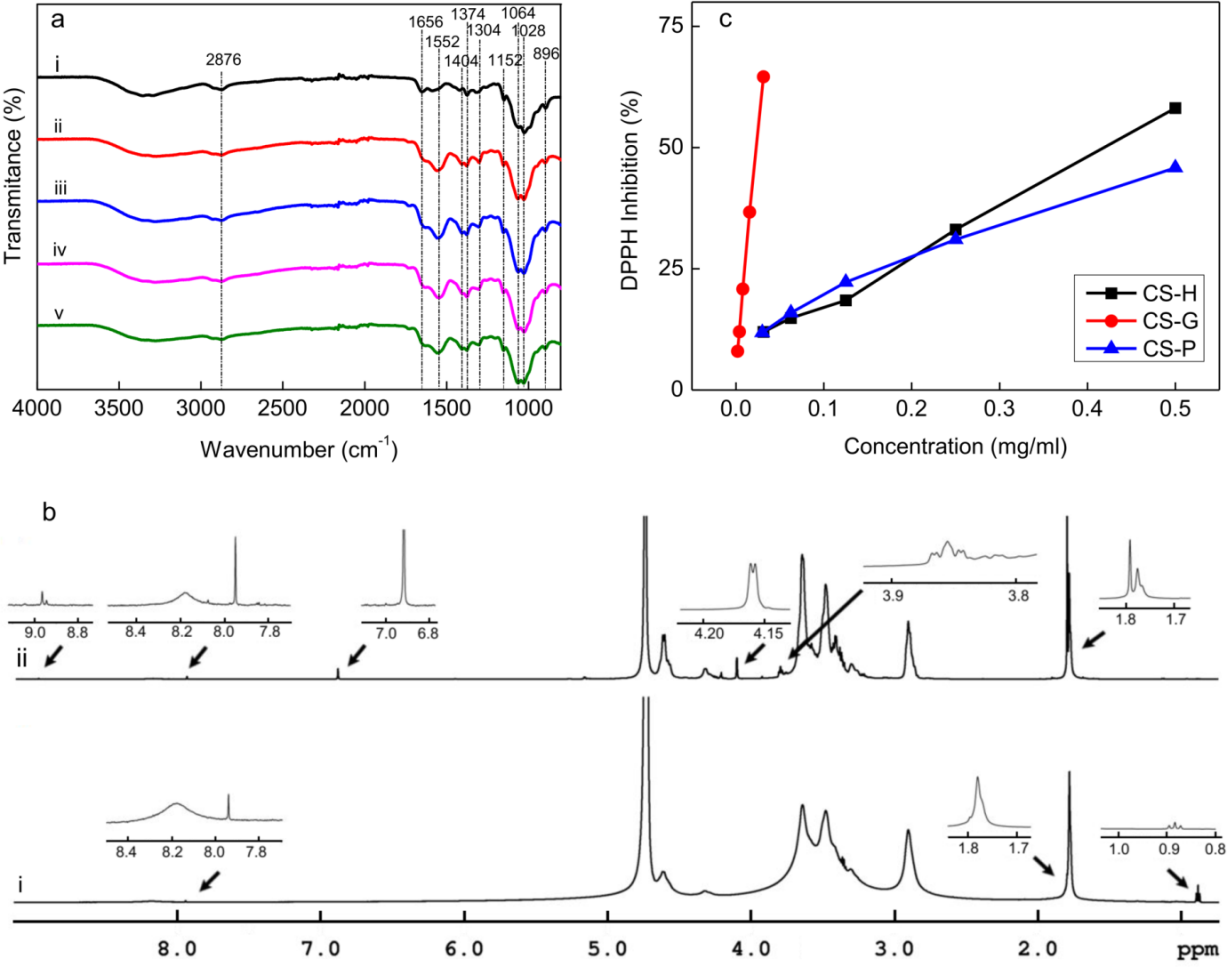
ATR-FTIR spectra of (a) chitosans: (i) precursor chitosan before modification, (ii) chitosan after degradation by the H_2_O_2_/ascorbic acid redox system and modification with (iii) gallic acid (CS-G), (iv) hydroquinone (CS-H), and (v) phloroglucinol (CS-P). (b) ^1^H NMR spectra of (i) CS and (ii) CS-G. (c) Dependence of DPPH inhibition on the concentration of modified chitosans.

The effect of the AA/H_2_O_2_ redox system and the phenolic compounds on the molecular structure of chitosan was investigated using ATR-FTIR and ^1^H NMR spectroscopy. The ATR-FTIR spectra of CS and of chitosans modified with phenolic compounds differed from that of the precursor chitosan. Two new peaks appeared at 1552 and 1728 cm^−1^ as a result of polymer degradation by the AA/H_2_O_2_ redox system (Figure 1a). Peaks in the range of 1500–1660 cm^−1^ were ascribed to amine or amide groups, namely, the peak at 1590 cm^−1^ was attributed to NH_2,_ and the peaks at 1660 and 1566 cm^−1^ were attributed to C=O and N–H bending vibrations of the amide group, respectively. Furthermore, the peak at 1728 cm^−1^ was attributed to C=O stretching. The appearance of these new peaks suggested that the hydroxy and amine groups of the precursor chitosan were sensitive to the action of radicals. In addition to the appearance of new bands, several peaks were shifted or changed in shape. The peaks at 1644 and 1426 cm^−1^ in the spectrum of the precursor chitosan were shifted to 1633 and 1404 cm^−1^, respectively, and the peak at 1064 cm^−1^ increased in intensity compared to the peak at 1028 cm^−1^. Peaks in the range of 1000–1150 cm^−1^ corresponded to C–O–C and C–O stretching of the glycoside linkage, and the peak at ca. 1400 cm^−1^ was ascribed to CH_3_ in the amide group. Other peaks at 896, 2876, and ca. 3338 cm^−1^ were attributed to breathing of the glucopyranose ring, C–H, and O–H stretching, respectively. In the ATR-FTIR spectra of chitosans modified with phenolic compounds, the peak at 1728 cm^−1^ was slightly shifted to 1734 cm^−1^, indicating binding of phenolic compounds to the carbonyl groups of CS.

Similar to ATR-FTIR spectra, the ^1^H NMR spectra of CS and chitosans modified with phenolic compounds resembled each other (Figure 1b). After free-radical grafting, new peaks appeared at 3.85, 3.98, 6.91 (only in CS-G), 8.85, and 9.19 ppm. Moreover, peaks at 1.78, 4.32, and 4.61 ppm changed their multiplicity and intensity. Peaks at 6.91, 8.85, and 9.19 ppm were ascribed to the 1H, 3OH, and 2OH protons of the aromatic benzene ring, respectively, confirming the binding of phenolic compounds to the chitosan [18].

According to the SEC results, treatment of the precursor chitosan with AA/H_2_O_2_ led to a significant decrease in the number-average (*M*_n_) and weight-average (*M*_w_) molecular weight of the polymer (Table 1). The values of *M*_n_ and *M*_w_ of the precursor chitosan were 2.5·10^3^ and 5.4·10^3^ kDa, respectively, i.e., *M*_w_/*M*_n_ = 2.09. The *M*_n_ values of CS-G and CS-H were lower (*M*_n_ = 7.33 and 7.73 kDa, respectively), and the highest *M*_n_ value was observed for CS-P (*M*_n_ = 8.45 kDa). The *M*_w_/*M*_n_ values of the latter polymers were ca. 35% lower than that of precursor chitosan, which indicated a lower polydispersity of the modified chitosans (Table 1).

**Table 1:** Number-average (*M*_n_) and weight-average (*M*_w_) molecular mass of chitosan before and after free-radical grafting with various phenolic compounds.

polymer	*M*_n_ (kDa)	*M*_w_ (kDa)	*M*_w_/*M*_n_

CS^a^	2.5·10^3^	5.4·10^3^	2.09
CS-G^b^	7.33	10.20	1.39
CS-H^c^	7.73	10.29	1.33
CS-P^d^	8.45	12.52	1.48

^a^CS: chitosan, ^b^CS-G: gallic acid-modified chitosan, ^c^CS-H: hydroquinone-modified chitosan, ^d^CS-P: phloroglucinol-modified chitosan.

**Scheme 1:**
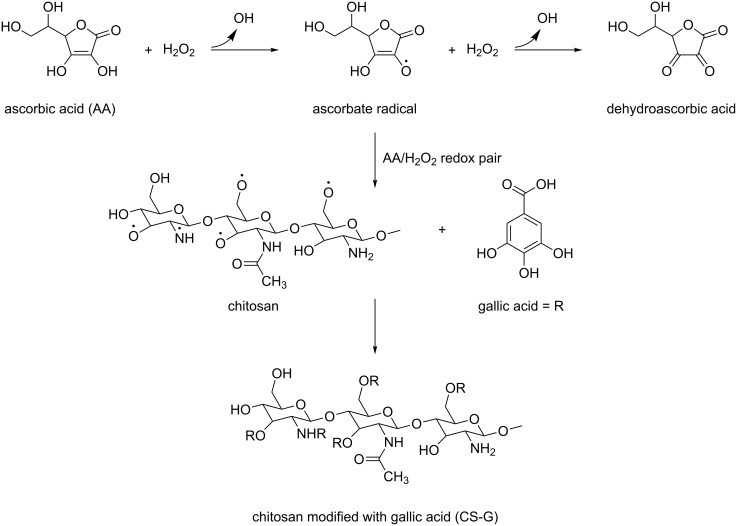
The proposed mechanism of the modification of chitosan by phenolic compounds exemplified with gallic acid.

Free radicals had two functions in the modification of chitosan: (i) the degradation of the polymer by chain scission and (ii) the creation of reactive sites accessible for the attachment of phenolic compounds. In free-radical grafting, the key role is played by hydroxyl radicals (^·^OH) that are generated through ascorbate radicals as a result of H_2_O_2_-induced oxidation of AA (Scheme 1). As the reaction proceeds at RT and no toxic byproducts are formed, the AA/H_2_O_2_ redox system is convenient for chitosan degradation [19–20]. Hydroxyl radicals attack the polysaccharide, forming macroradicals, which are then prone to covalently bind phenolic compounds. According to the ATR-FTIR and ^1^H NMR spectroscopy results, the structural similarity between CS and chitosans modified with phenolic compounds was confirmed. Due to the presence of primary amino groups (p*K*_a_ = 6.3), chitosans with high and medium molecular weight are water-insoluble, but they can be readily dissolved under mild acidic conditions [21]. After free-radical grafting, both *M*_n_ and *M*_w_ of the chitosans modified with phenolic compounds notably decreased, reaching approximately the same values, which was accompanied by a reduction in the viscosity of the solution. Based on the results, it was assumed that the molecular structure of the modified chitosans was mainly determined by the method chosen to attach phenolic compounds on the chitosan rather than by the phenolic compounds themselves. Moreover, the chitosans with low molecular weight were highly soluble in 2 wt % acetic acid; they could also be dissolved in water, which is important for surface modification of the magnetic particles.

#### Antioxidant properties of chitosans modified with phenolic compounds

The FC assay for colorimetric determination of total phenolic content in samples of natural origin is based on the electron transfer between a phenolic compound and a phosphomolybdic/phosphotungstic complex that emits at a wavelength of 765 nm [22]. The FC reagent measures the total antioxidant capacity of not only phenolic compounds but also carbohydrates, thiols, vitamins, amines, unsaturated fatty acids, and various complex specimens [23]. The antioxidant properties of the modified chitosans increased in the following sequence: CS-H < CS-P < CS-G (Table 2). The total phenolic content in CS-H reached 5.4 mg/g, which corresponds to 50% of the value achieved for CS-P (10.8 mg/g). Compared to CS-P, the total phenolic content of CS-G increased by another 8 mg/g to 18.8 mg/g in total.

**Table 2:** Antioxidant properties of chitosans modified with phenolic compounds.

	CS-G	CS-H	CS-P

GAE (mg/g)^a^	18.8 ± 0.2	5.4 ± 0.2	10.8 ± 0.4
IC_50_ (mg)^b^	0.023 ± 0.001	0.564 ± 0.016	0.492 ± 0.012

^a^GAE: gallic acid equivalent, ^b^IC_50_: half maximal inhibitory concentration.

According to the DPPH assay, the radical scavenging capacity of CS-G, expressed as the IC_50_ value, dramatically differed from that of the rest of the modified chitosans (Table 2; Figure 1c). The IC_50_ value of CS-G (0.023 mg/mL) was one order of magnitude smaller than that of CS-P and CS-H (0.564 and 0.492 mg/mL, respectively). As a result, the ability of the modified chitosans to scavenge DPPH radicals increased in the same order as observed in the FC assay.

The antioxidant properties of the chitosans increase with an increasing degree of deacetylation and a decreasing molecular weight. Chitosan from crab shells exhibits good antioxidant properties, defined as the ability to scavenge hydroxyl radicals and to chelate ferrous ions [14]. Because the chitosans modified with phenolic compounds were characterized by a low *M*_n_, it was assumed that their antioxidant properties originated either from the polymeric or phenolic compounds. However, due to the similar molecular weights of the modified chitosans, differences in antioxidant properties were ascribed to the phenolic compounds. According to both the FC and DPPH assays, the highest antioxidant activity was observed for CS-G, while the lowest antioxidant activity was observed for CS-H. Moreover, the radical scavenging ability of CS-P was closer to that of CS-H than that of CS-G. The antioxidant properties of hydroquinone, which has only two hydroxy groups attached to the aromatic ring, were relatively low, whereas those of gallic acid, which has three hydroxy groups and a carbonyl group, were the highest. It can be explained by the superior stability of a gallic acid-derived free radical, which is a decisive factor in the antioxidant capacity of phenolic compounds. In contrast to hydroquinone and phloroglucinol, gallic acid is able to both donate electrons from hydroxy groups and hydrogen atoms from carboxyl groups and these two mechanisms contribute to the resonance stabilization of the resulting radical. However, the significant qualitative differences between the antioxidant properties of CS-G and other chitosans modified with phenolic compounds according to the FC and DPPH assays indicated the necessity of using additional methods for the examination of the antioxidant properties.

#### γ-Fe_2_O_3_ nanoparticles

**Figure 2:**
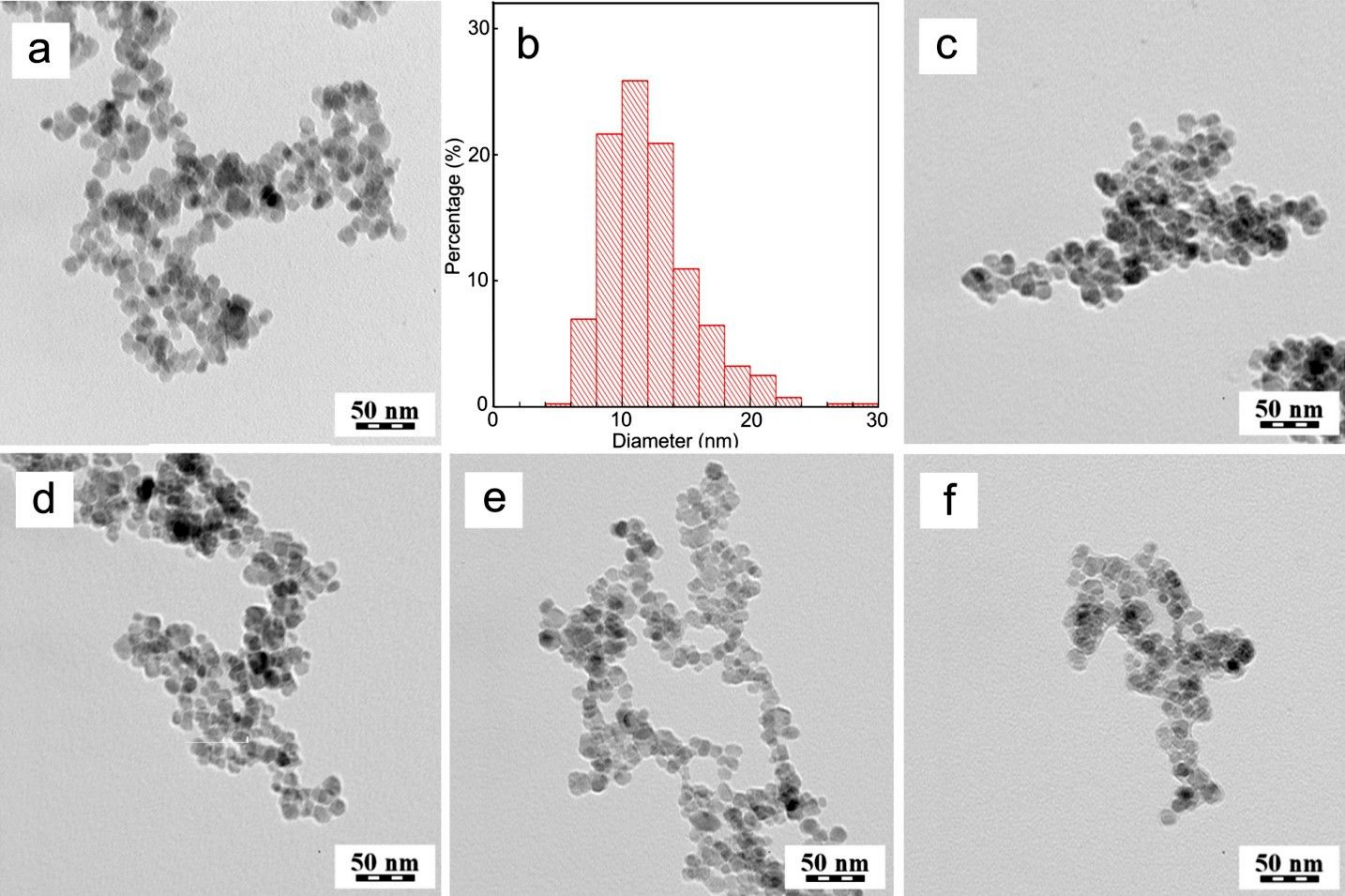
TEM micrographs of (a) γ-Fe_2_O_3_, (c) γ-Fe_2_O_3_@Hep, (d) γ-Fe_2_O_3_@Hep-CS-H, (e) γ-Fe_2_O_3_@Hep-CS-G, and (f) γ-Fe_2_O_3_@Hep-CS-P nanoparticles. (b) Size-distribution histogram of γ-Fe_2_O_3_ nanoparticles.

The advantages of iron oxides in biomedical applications include biocompatibility, excellent magnetic properties, and the possibility to modify the surface with reactive functional groups. In this study, magnetite (Fe_3_O_4_) nanoparticles were synthetized by coprecipitation of iron(II) chloride and iron(III) chloride with ammonia and subsequently oxidized with hydrogen peroxide under mild acidic conditions. The resulting maghemite (γ-Fe_2_O_3_) has the benefit of higher chemical stability over Fe_3_O_4_ due to the lack of iron(II) atoms that are prone to oxidation [24]. TEM micrographs of the γ-Fe_2_O_3_ particles showed their quasi-spherical shape and a moderately broad size distribution (Figure 2a). The dispersity of the particles (*Ð*) was 1.25, and the number-average particle diameter (*D*_n_) was 11 nm (Figure 2b).

As expected, the hydrodynamic diameter of γ-Fe_2_O_3_ in water (*D*_h_ = 91 nm) was substantially larger than the *D*_n_ value of the dry particles, as it consists of both nanoparticle core and solvation layer (Table 3). Moreover, *D*_h_ represents the Z-average particle diameter, which is proportional to the sixth power of size and, in contrast to *D*_n_, is more sensitive to the presence of large objects. The polydispersity of γ-Fe_2_O_3_ (PD = 0.33), which characterizes the width of the particle size distribution, was relatively high, indicating the presence of particle agglomerates (doublets, triplets and small clusters), due to the absence of a polymeric steric stabilizer, and of larger particles, which was also confirmed by TEM microscopy (Figure 2b). The ζ-potential of the γ-Fe_2_O_3_ particles was positive (ca. 50 mV) due to the presence of cations on the particle surface, originating from oxidation under mildly acidic conditions. This high ζ-potential value is considered to be more than sufficient to ensure good colloidal stability of the particles in water.

**Table 3:** Nanoparticle characterization by DLS.

nanoparticles	*D*_h_ (nm)	PD	ζ-potential (mV)

γ-Fe_2_O_3_	91	0.33	48
γ-Fe_2_O_3_@Hep	60	0.18	−60
γ-Fe_2_O_3_@Hep-CS-G	81	0.11	30
γ-Fe_2_O_3_@Hep-CS-H	81	0.11	29
γ-Fe_2_O_3_@Hep-CS-P	90	0.12	26

**Figure 3:**
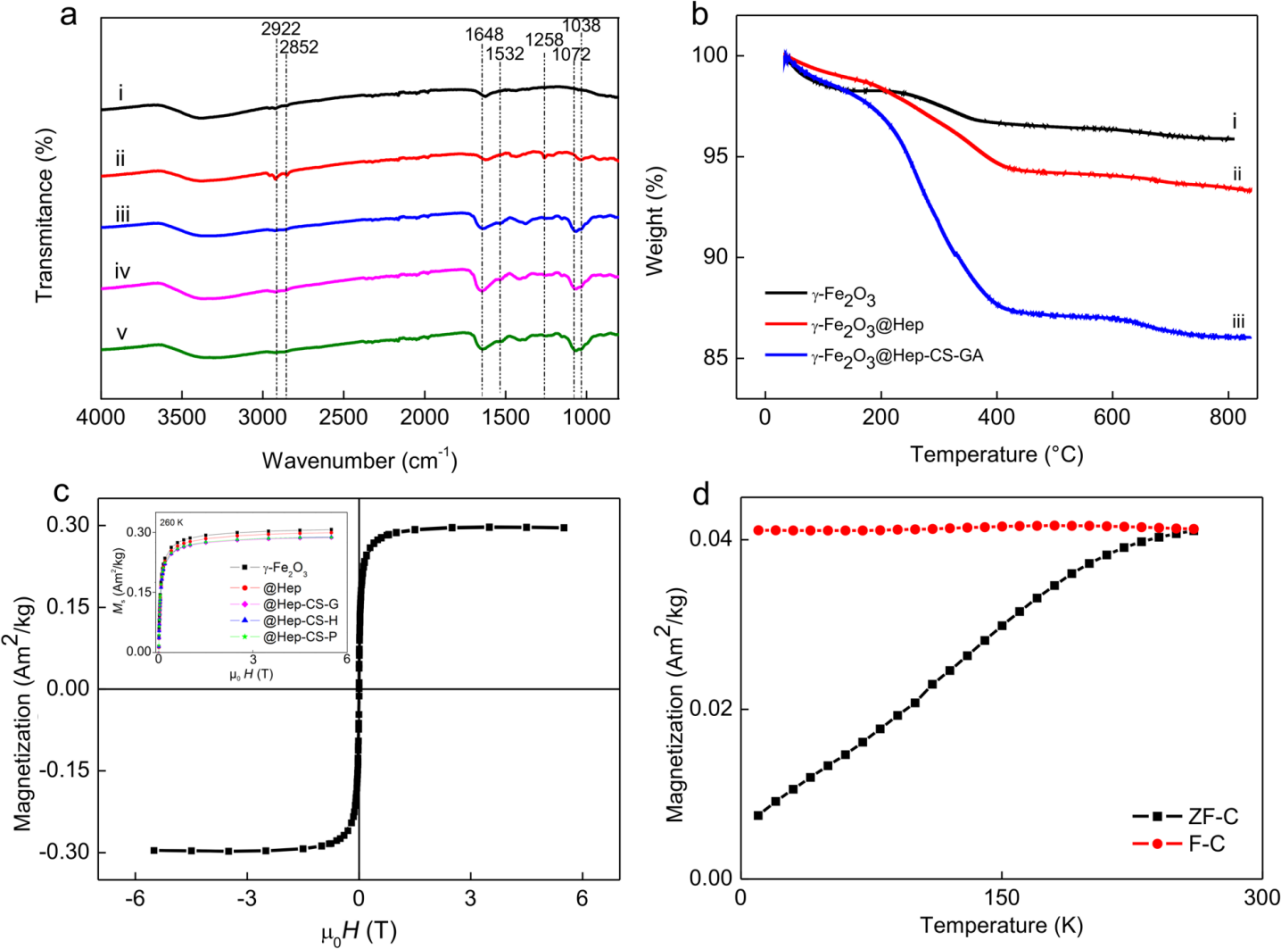
(a) ATR-FTIR spectra of (i) γ-Fe_2_O_3_, (ii) γ-Fe_2_O_3_@Hep, (iii) γ-Fe_2_O_3_@Hep-CS-G, (iv) γ-Fe_2_O_3_@Hep-CS-H, and (v) γ-Fe_2_O_3_@Hep-CS-P nanoparticles. (b) TGA of (i) γ-Fe_2_O_3_, (ii) γ-Fe_2_O_3_@Hep, and (iii) γ-Fe_2_O_3_@Hep-CS-G. (c) Magnetic hysteresis loop of the aqueous γ-Fe_2_O_3_ colloid (4.4 mg/mL) at 260 K. The inset shows the magnetization curves of all colloids normalized by the nanoparticle weight. (d) Temperature dependence of the magnetization of the γ-Fe_2_O_3_@Hep-CS-G colloid. *H* is the applied magnetic field, and μ_0_ is the magnetic permeability of vacuum.

ATR-FTIR spectra were measured in the range of 800–4000 cm^−1^. The majority of the absorption peaks associated with the vibration of γ-FeO were located below this range, and only one weak peak at ca. 896 cm^−1^ was ascribed to pure γ-Fe_2_O_3_ [25]. The absorption peak above 3000 cm^−1^ corresponds to the O–H stretching vibration (Figure 3a).

According to the TGA results, the γ-Fe_2_O_3_ weight loss occurred in two temperature ranges (Figure 3b). In the first temperature range (35–200 °C), the weight loss was 1.7 wt %, while in the second temperature range (200–400 °C), the weight loss was 1.6 wt % The total weight loss up to 800 °C was 4.1 wt %, which was mainly attributed to the removal of residual water and water that was bound to γ-Fe_2_O_3_.

The superparamagnetism of the γ-Fe_2_O_3_ colloid was confirmed by SQUID magnetometry through the absence of remanence and coercivity in the magnetic hysteresis curve (Figure 3c,d). The saturation magnetization of the γ-Fe_2_O_3_ colloid (4.4 mg/mL was 0.307 A·m^2^·kg^−1^ at 260 K. The critical parameter for the magnetism of nanoparticles is the particle size. γ-Fe_2_O_3_ nanoparticles with sizes below the single-domain critical diameter are superparamagnetic, whereas larger particles are ferrimagnetic [26–27]. Superparamagnetism is an important feature of magnetic nanoparticles intended for biomedical applications, because superparamagnetic particles behave as nonmagnetic materials in the absence of a magnetic field, and consequently, aggregation of the nanoparticles induced by magnetic forces is minimized.

#### Heparin-coated γ-Fe_2_O_3_ nanoparticles

The role of the heparin layer is to isolate the inorganic core from the phenolic compounds and allow for the attachment of the cationic polymer (chitosan). Heparin is a polysaccharide, containing glycosaminoglycan with densely repeated *O*-(α-L-iodopyranosyluric acid 2-sulfate)-(1→4)-2-sulfoamino-2-deoxy-D-glucose 6-sulfate sequences obtained from the mucosal tissue of animals, such as pigs and cattle [28]. Unfractionated and low-molecular-weight heparins are used in medicine as indirect anticoagulants for thrombosis prevention and treatment [29]. The presence of sulfate and carboxyl groups in heparin was critical for surface modification of the positively charged γ-Fe_2_O_3_ nanoparticles via the LbL technique. Heparin was attached to the γ-Fe_2_O_3_ surface via electrostatic interactions.

The TEM micrograph of the γ-Fe_2_O_3_@Hep particles showed the heparin layer as a thin bright halo around the iron oxide core (Figure 2c). Compared to γ-Fe_2_O_3_, the *D*_h_ value of the γ-Fe_2_O_3_@Hep particles decreased from 91 to 60 nm, the polydispersity (PD) decreased from 0.33 to 0.18, and the absolute value of the ζ-potential increased from 48 to 60 mV (Table 3). The decrease in the *D*_h_ and PD values indicated that heparin served as a good stabilizer of the γ-Fe_2_O_3_ nanoparticles, hindering their aggregation. The stabilizing effect of the heparin layer was also indicated by the changes in the ζ-potential of the particles before and after modification. Because the absolute value of the ζ-potential is a measure of the repulsion forces between particles in the medium, it indicates the colloidal stability of the dispersion. A higher absolute value of the ζ-potential indicates stronger repulsion forces and greater colloidal stability. Moreover, the change in the ζ-potential from positive to negative values indicated that heparin was successfully attached to the particle surface.

In the ATR-FTIR spectrum of the γ-Fe_2_O_3_@Hep particles, several new peaks were observed at 2922, 2852, 1258, and 1038 cm^−1^ compared to the γ-Fe_2_O_3_ spectrum (Figure 3a). Peaks at 2922, 2852, and 1648 cm^−1^ were ascribed to C–H and C=O stretching vibrations of carboxyl groups. Peaks at 1258 and 1038 cm^−1^ corresponded to N–H and C–O stretching vibrations of the amine groups and polysaccharides, respectively.

The similarity between the magnetic hysteresis of neat and heparin-modified nanoparticles indicated that the polymer-coated nanoparticles retained the same superparamagnetic behavior. However, a small decrease in the saturation magnetization of the γ-Fe_2_O_3_@Hep colloid (by 2%) at 260 K was observed compared to the saturation magnetization of γ-Fe_2_O_3_ at the same particle concentration (inset in Figure 3c). This decrease was caused by the increased content of the nonmagnetic fraction in the particles.

The TGA result of the γ-Fe_2_O_3_@Hep nanoparticles significantly differed from that of the γ-Fe_2_O_3_, especially in the range of 200–400 °C (Figure 3b). While the weight loss of the γ-Fe_2_O_3_ nanoparticles was mostly caused by evaporation of water, the weight loss of the γ-Fe_2_O_3_@Hep was associated with decomposition of heparin on the particle surface. The weight loss of γ-Fe_2_O_3_@Hep particles in the range of 35–400 °C was 6.4 wt %, which was nearly double of that observed for the neat γ-Fe_2_O_3_ particles. The total γ-Fe_2_O_3_@Hep weight loss was 6.5 wt %, including ca. 2.5 wt % loss that was ascribed to heparin.

#### γ-Fe_2_O_3_@Hep nanoparticles coated with modified chitosan

The second layer on the γ-Fe_2_O_3_ particles consisted of one of the chitosans modified with phenolic compounds. The TEM micrographs showed no sharp boundary between particles with one and two polymer layers (Figure 2d–f). The organic layer was visible as a bright halo of nanometer thickness around the iron oxide core. Because both polymers (heparin and modified chitosan) had similar elemental compositions and densities, it was impossible to distinguish between them in the TEM micrographs.

The introduction of the second layer significantly increased the *D*_h_ value of the modified chitosan-coated particles (Table 3). The *D*_h_ value of the γ-Fe_2_O_3_@Hep-CS-G and γ-Fe_2_O_3_@Hep-CS-H particles was 81 nm, i.e., 20 nm larger than that of the γ-Fe_2_O_3_@Hep particles. The hydrodynamic diameter of the γ-Fe_2_O_3_@Hep-CS-P particles (*D*_h_ = 90 nm) was similar to that of the neat particles. The increase in *D*_h_ was accompanied by a decrease in the polydispersity (PD = 0.11), a decrease in the absolute ζ-potential value to ca. 28 mV, and a change in the ζ-potential value from negative to positive. These characteristics suggested that the modified chitosans were attached to the heparin layer via electrostatic interactions. The relatively low ζ-potential of the particles compared to that of γ-Fe_2_O_3_ and γ-Fe_2_O_3_@Hep indicated that the repulsion forces between the particles in water were weaker.

The ATR-FTIR spectra of the particles modified with the second (chitosan) layer were similar to those of the modified chitosans; however, the peaks were slightly shifted to lower values. For example, peaks at 1038 and 1072 cm^−1^ in the spectra of the modified chitosan-coated particles corresponded to the peaks at 1028 and 1064 cm^−1^ in the spectrum of the modified chitosans (Figure 3a), respectively.

Additionally, the TGA thermograms of the phenolic compound-coated γ-Fe_2_O_3_ particles were similar to those of the γ-Fe_2_O_3_ and γ-Fe_2_O_3_@Hep particles, confirming that the type of phenolic compound bound to the chitosan did not affect the thermal properties of the particles (Figure 3b). Particles coated with the phenolic compound-modified chitosans had the highest weight loss at temperatures ranging from 200 to 420 °C. The total weight loss of γ-Fe_2_O_3_@Hep-CS-H, γ-Fe_2_O_3_@Hep-CS-G, and γ-Fe_2_O_3_@Hep-CS-P particles was 9.8, 12.1, and 10.9 wt %, respectively. The content of the phenolic compound-modified chitosan on the particles varied in the range of 3.3–5.7 wt % and was the highest for γ-Fe_2_O_3_@Hep-CS-G and the lowest for γ-Fe_2_O_3_@Hep-CS-H. Therefore, the structure of the phenolic compound bound to the chitosan only slightly affected the coating efficiency.

The magnetic behavior of the chitosan-modified particles did not change compared to that of the γ-Fe_2_O_3_ colloid. As expected, the magnetic saturation decreased with increasing content of the nonmagnetic organic phase at the nanoparticle surface, which was previously verified for the γ-Fe_2_O_3_@Hep colloid. At the same concentration, the saturation magnetization values of the chitosan-modified colloids at 260 K were similar and lower than those of the γ-Fe_2_O_3_ (by 6.3%) and γ-Fe_2_O_3_@Hep colloids (by 4.2%). Measurements of the magnetization as a function of the temperature were also performed for all colloids. The curves were very similar to that for the γ-Fe_2_O_3_@Hep-CS-G (Figure 3d). The field-cooled (F-C) curve remained approximately constant, and the zero-field-cooled (ZF-C) curve slowly increased up to 250 K, indicating that the nanoparticles were in a blocked state below this temperature but were relaxed above this temperature. This confirmed their genuine superparamagnetic behavior at room temperature [30].

**Figure 4:**
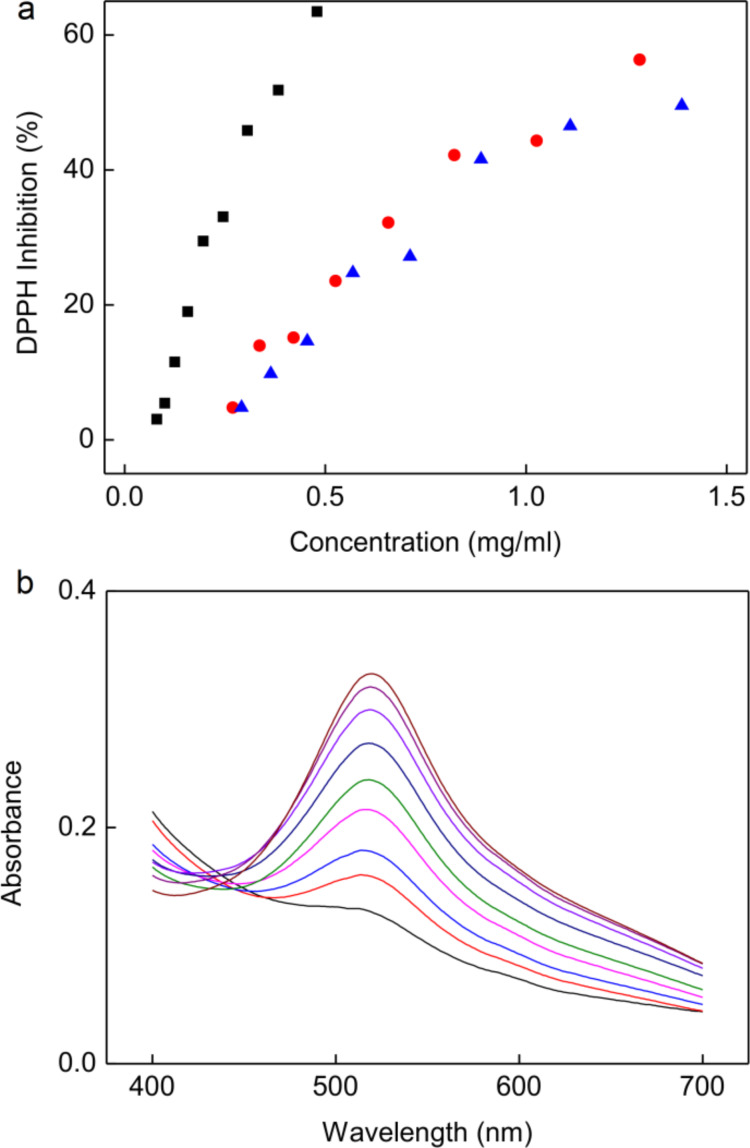
Free radical scavenging of the phenolic compound-modified particles. (a) Inhibition as a function of the concentration of γ-Fe_2_O_3_@Hep-CS-G (black squares), γ-Fe_2_O_3_@Hep-CS-H (red circles), and γ-Fe_2_O_3_@Hep-CS-P particles (blue triangles) according to the DPPH assay and (b) absorbance of DPPH solution with the γ-Fe_2_O_3_@Hep-CS-G colloid of different concentrations (0.08–0.50 mg/mL); DPPH: 2,2-diphenyl-1-picrylhydrazyl.

Because the modified chitosans exhibited antioxidant properties due to the presence of antioxidant molecules, it was expected that phenolic compound-modified particles would also demonstrate high effectiveness in removing free radicals. Among the particles, the γ-Fe_2_O_3_@Hep-CS-G particles showed the highest free radical scavenging potential (IC_50_ = 0.53 ± 0.08 mg/mL; Figure 4a). The concentrations of the γ-Fe_2_O_3_@Hep-CS-H (1.00 ± 0.08 mg/mL) and γ-Fe_2_O_3_@Hep-CS-P particles (1.10 ± 0.08 mg/mL) that were required to decrease the number of radicals by half were two times greater than that of γ-Fe_2_O_3_@Hep-CS-G. Therefore, similar to chitosans modified with phenolic compounds, the γ-Fe_2_O_3_@CS-P and γ-Fe_2_O_3_@CS-H particles also had analogous antioxidant properties, and the antioxidant activity of the γ-Fe_2_O_3_@CS-G particles was the highest. However, the difference between the radical scavenging ability of the phenolic compound-modified particles was not so pronounced compared to that of the phenolic compound-modified chitosans. Even so, γ-Fe_2_O_3_@CS-H and γ-Fe_2_O_3_@CS-P particles showed higher scavenging capacity than expected based on the results of the DPPH test of the chitosans (Figure 1c) and TGA analysis of the particles (Figure 3b). The high weight of the inorganic core contributed greatly to the IC_50_ value expressed in milligrams per milliliter, while the phenolic compound-modified chitosans constituted only up to 5.7% of the total weight of the prepared particles. The obtained results indicated that in case of the lack of sufficient antioxidant capacity of polymer coating, further reactions occur on the surface of the particle core. In addition, polymer intermixing, the configuration of hydroxy groups on the benzene rings, and especially particle aggregation, which is associated with limitation of accessible surface, can also influence the final antioxidant activity [31].

#### Cellular uptake of the nanoparticles

**Figure 5:**
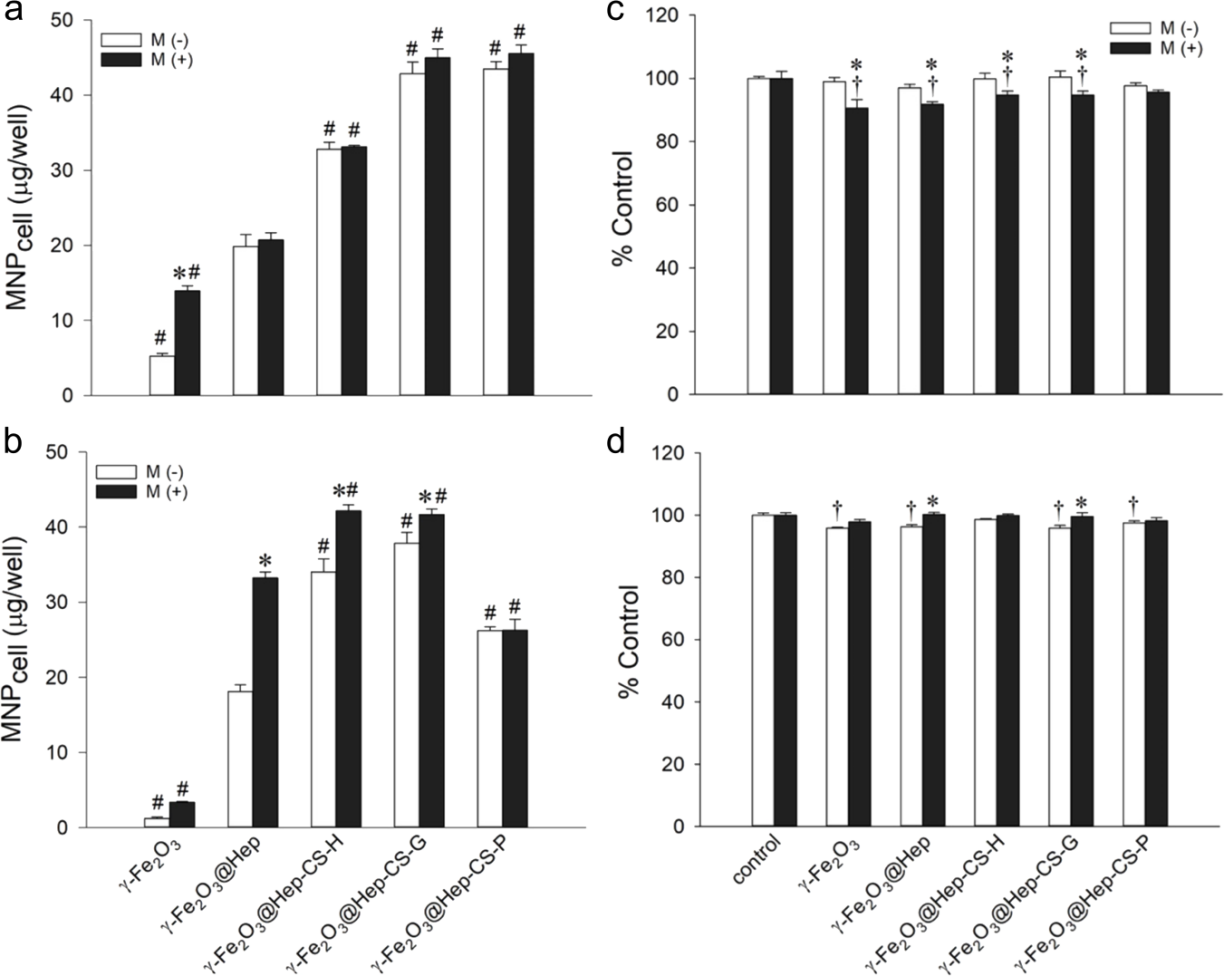
Cellular uptake of the phenolic compound-modified particles by (a) L-929 and (b) LN-229 cells. A magnetic field was applied for 5 min, M (−), or 3 h, M (+), after administration of the particles (100 μg/mL). Values are shown as the mean ± standard error (SE; *n* = 3). *^,^**^#^** *p* < 0.05 compared to the corresponding M (−) and γ-Fe_2_O_3_@Hep species, respectively. Cell viability of the phenolic compound-modified particles in (c) L-929 and (d) LN-229 cells. A magnetic field was applied for 5 min, M (−) or 3 h, (M +), after administration of the particles (100 μg/mL). The control measurement was performed in the absence of the particles. Values are shown as the mean ± SE (*n* = 3). *^,†^ *p* < 0.05 compared to the corresponding M (−) and control groups, respectively.

To determine the effect of the phenolic compound-modified nanoparticles on modulating intracellular ROS levels, the amount of the cell-associated nanoparticles (MNP_cell_) and cell viability were determined after incubation of γ-Fe_2_O_3_, γ-Fe_2_O_3_@Hep, γ-Fe_2_O_3_@Hep-CS-G, γ-Fe_2_O_3_@Hep-CS-H, or γ-Fe_2_O_3_@Hep-CS-P nanoparticles (100 μg/mL) with L-929 or LN-229 cells for 3 h (Figure 5a,b). In the absence of a magnetic field, the heparin coating enhanced the MNP_cell_ value to 3.8- and 14.9-fold greater than that without heparin in L-929 and LN-229 cells, respectively. The results were consistent with previous findings indicating that heparin modification may increase the cellular uptake of nanoparticles by the interaction of heparin with the growth factor receptor on the cell membrane [32–33]. Particles with phenolic modification further increased the MNP_cell_ value to 1.7- to 2.2-fold and 1.4- to 2.1-fold greater values in L-929 and LN-229 cells, respectively, compared to that of γ-Fe_2_O_3_@Hep. These results indicated that phenolic modification may further augment particle internalization compared to heparin modification alone. Previous studies have suggested that electrostatic interactions, oxidation-dependent conjugation, and hydrogen bonds between the phenolic OH groups and polysaccharide moieties and/or amino acid residues of the cell membrane may enhance nanoparticle internalization [34].

The application of a magnetic field during incubation with γ-Fe_2_O_3_ increased the MNP_cell_ level by 2.7-fold compared with that without the magnet in L-929 cells. The γ-Fe_2_O_3_@Hep uptake in LN-229 cells was increased by 1.8-fold compared with that without magnetic field. However, the application of a magnetic field exerted either no increase or a minor increase in the MNP_cell_ value of the phenolic compound-modified nanoparticles in L-929 or LN-229 cells, suggesting that phenolic modification may facilitate uptake to a level near the maximum uptake capacity. Our results were consistent with previous findings indicating that the application of a magnetic field did not facilitate cellular uptake of the magnetic nanoparticles [35–36].

The cytotoxicity of the nanoparticles (100 μg/mL) after 3 h of incubation with L-929 and LN-229 cells was not significant or very minor (Figure 5c,d). The viability of the cells treated with the nanoparticles remained within 91–100% compared to the control cells in both cell types regardless of the presence or absence of a magnetic field.

#### ROS scavenging activity of the nanoparticles

**Figure 6:**
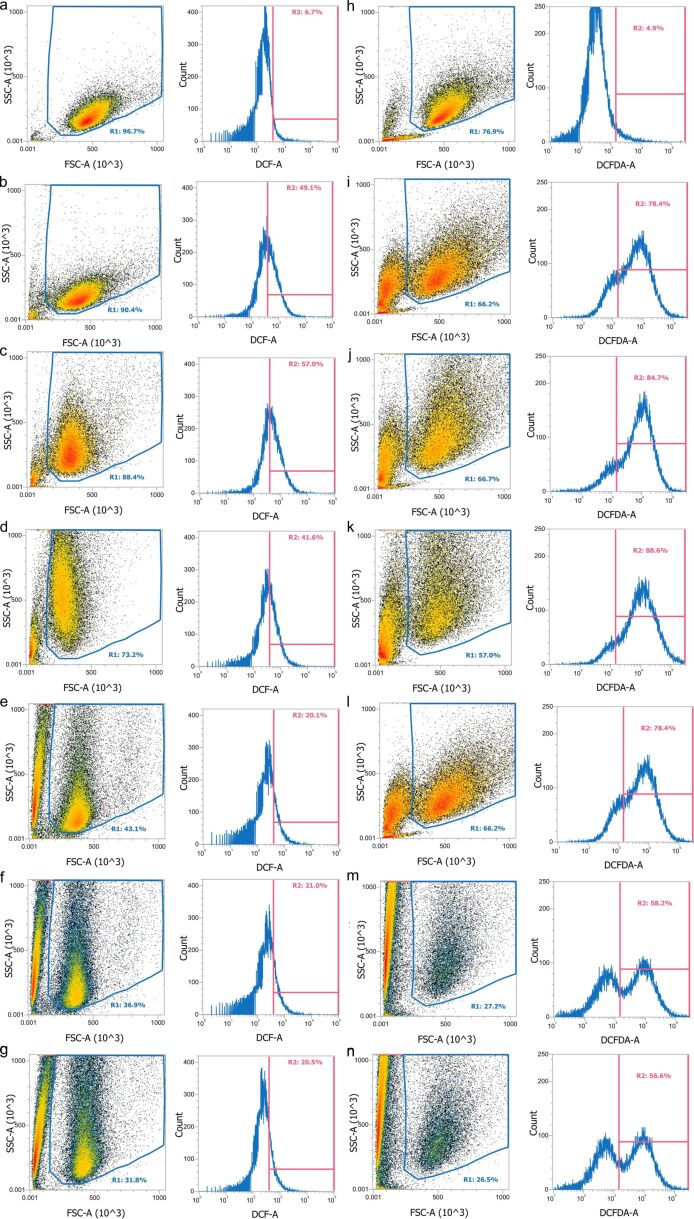
Representative antioxidant activity of the phenolic compound-modified particles in (a–g) L-929 and (h–n) LN-229 cells. The cells were incubated with (c, j) γ-Fe_2_O_3_, (d, k) γ-Fe_2_O_3_@Hep, (e, l) γ-Fe_2_O_3_@Hep-CS-G, (f, m) γ-Fe_2_O_3_@Hep-CS-H, and (g, n) γ-Fe_2_O_3_@Hep-CS-P particles for 3 h, followed by 30 min incubation with 2 mM H_2_O_2_ (b–g, i–n) prior to analysis using flow cytometry. (a, h) Only cells without and (b, i) with H_2_O_2_ treatment.

**Figure 7:**
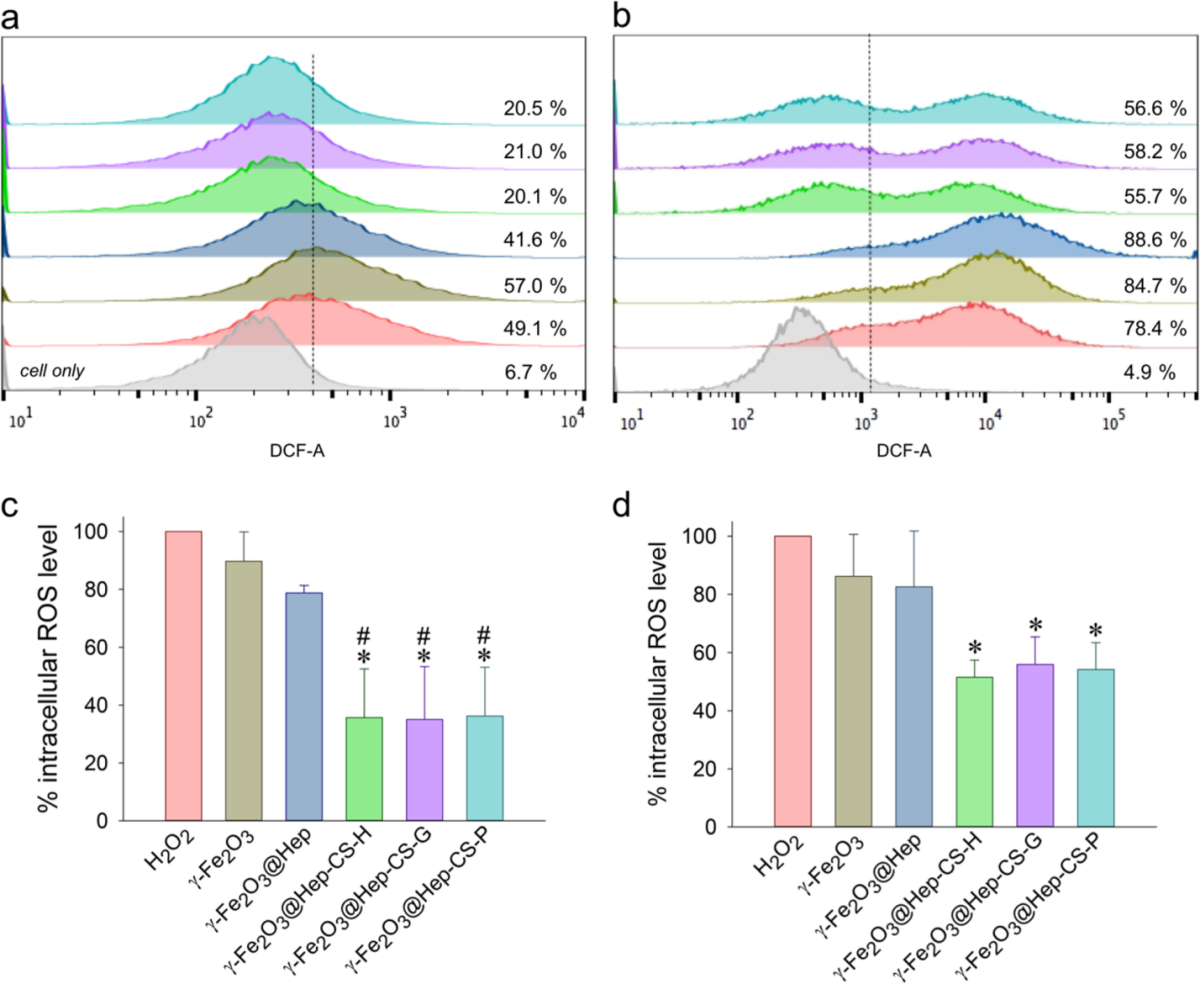
Flow cytometry analysis of the antioxidant activity of the phenolic compound-modified particles based on intracellular ROS levels in (a, c) L-929 and (b, d) LN-229 cells. (a, b) The representative fluorescence shift was derived from Figure 6. (c, d) Intracellular ROS levels were normalized to the H_2_O_2_ levels. *^,^**^#^** *p* < 0.05 compared to the corresponding results with H_2_O_2_ and γ-Fe_2_O_3_@Hep, respectively. Values are shown as the mean ± SE (*n* = 3–4).

To determine the ROS scavenging activity of the phenolic compound-modified nanoparticles (100 μg/mL), they were incubated with L-929 and LN-229 cells for 3 h, and 2 mM H_2_O_2_ was added for 30 min, followed by staining with CM-H_2_DCFDA for 1 h. Figure 6 shows the representative flow cytometry results of nanoparticle internalization and the intracellular ROS levels after treatment with hydrogen peroxide. The density plot in the left panel shows the relationship between cellular volume and complexity. The R1 region in the density plot indicated cell population, whereas the left population outside the R1 region was related to the nanoparticles loosely bound on the cell surface or cell debris. After incubation with the nanoparticles, the upper shifted cell population in the R1 region indicated an increase in cellular complexity (Figure 6c–g, Figure 6j–n), suggesting nanoparticle internalization. H_2_O_2_ treatment induced a right-shift of the DCF-A signal, suggesting an increase in the cellular ROS level. Compared to non-treated cells (Figure 6a,h), treatment with H_2_O_2_ affected cell viability and resulted in an increase in cell debris (Figure 6b,i). The internalized phenolic compound-modified nanoparticles reduced the cellular ROS level significantly in both L-929 and LN-229 cells (Figure 6). The representative results are summarized in Figure 7a,b. Compared to γ-Fe_2_O_3_, the phenolic compound-modified nanoparticles reduced the cellular ROS level in L-929 cells to 20–21% from a basal level of 49% (Figure 7a), whereas the cellular ROS level in LN-229 cells was reduced from 78% to 55–58% (Figure 7b). Figure 7c,d summarizes multiple experimental results in L-929 and LN-229 cells, respectively, demonstrating a significant decrease in intracellular ROS levels by the phenolic compound-modified nanoparticles. Previous studies have reported that functional groups in the ortho-position of phenol are more active than groups in the *para*- and *meta*-positions. According to the literature [37] and this study, although gallic acid had superior antioxidant properties among the investigated phenols (Figure 1c), modification of γ-Fe_2_O_3_ with the three phenolic compounds exerted no difference on the ROS scavenging activity. The results suggested that phenolic compounds immobilized on the γ-Fe_2_O_3_ nanoparticles preserved their antioxidant effect to reduce ROS levels inside the cells.

## Conclusion

Magnetic nanoparticles are increasingly used for biomagnetic separation, targeting tumor sites using an external magnetic field, MRI contrast agents, or magnetic hyperthermia. Under physiological conditions, the nanoparticle surface is exposed to the action of biomolecules, oxygen, peroxides, and radicals, which changes particle properties and behavior. Moreover, Fe^2+^ ions can induce oxidative stress in living organisms via the Fenton reaction. Therefore, it is crucially important in biological applications of iron oxide nanoparticles to counteract this undesirable effect by antioxidants. The combination of magnetic targetability with antioxidant properties is an interesting field with many potential therapeutic applications.

In this study, superparamagnetic γ-Fe_2_O_3_ nanoparticles were coated with heparin and chitosans modified with different phenolic compounds and thoroughly characterized. The variation of the magnetization of the neat nanoparticles as a function of both temperature and magnetic field showed a typical superparamagnetic behavior of the γ-Fe_2_O_3_ nanoparticles with a saturation magnetization of 0.307 A∙m^2^·kg^−1^ at 260 K. The similarity of the magnetic hysteresis and ZF-C/F-C curves of the neat and modified nanoparticles indicated that the coatings did not affect the magnetic behavior. γ-Fe_2_O_3_ nanoparticles coated with phenolic compound-modified chitosans significantly promoted cellular uptake and ROS scavenging activity compared to γ-Fe_2_O_3_@heparin particles in both cell types that were investigated. Although the gallic acid-modified nanoparticles exerted the most potent free radical scavenging activity measured by using the DPPH assay, all three phenolic compound-modified nanoparticles reduced intracellular ROS levels in a similar manner as the control, as indicated by flow cytometry. Because intracellular oxidative stress has been shown to be elevated in many disorders, including neurodegenerative, malignant and cardiovascular diseases, these particle characteristics may be applicable in many theranostic areas without provoking ROS-related toxicity intrinsic to iron oxide particles.

## Experimental

### Materials

FeCl_2_·4H_2_O, FeCl_3_·6H_2_O, benzene-1,4-diol (hydroquinone), benzene-1,3,5-triol (phloroglucinol), Folin–Ciocalteu (FC) reagent, 2,2-diphenyl-1-picrylhydrazyl (DPPH) assay, precursor chitosan (highly viscous) from crab shells, heparin sodium salt (Hep) from porcine intestinal mucosa, hydrochloric acid, ammonium persulfate, potassium thiocyanate, hydrogen peroxide, and cell counting kit-8 (CCK-8) were purchased from Sigma-Aldrich (Milwaukee, WI, USA). Ammonium hydroxide (30%) and glacial acetic acid were obtained from Lach-Ner (Neratovice, Czech Republic). Sodium carbonate was purchased from Lachema (Neratovice, Czech Republic). 3,4,5-Trihydroxybenzoic acid (gallic acid) was purchased from Loba Feinchemie (Fischamend, Austria). Dulbecco’s modified Eagle’s medium, minimum essential medium Eagle, sodium pyruvate, antibiotic-antimycotic solution (penicillin/streptomycin/amphotericin B), and 6-carboxy-2′,7′-dichlorohydrofluorescein diacetate (DCFH-DA) were purchased from Thermo Fisher Scientific (Waltham, MA, USA). Fetal bovine serum and donor equine serum were purchased from HyClone Laboratories (Logan, UT, USA). All chemicals were used as received. Ultrapure water (Q-water), obtained using a Milli-Q Gradient A10 system (Millipore; Molsheim, France), was used during the synthesis and modification of the magnetic nanoparticles.

### Modification of chitosan with phenolic compounds

Chitosan derivatives with covalently attached phenolic compounds were prepared by free-radical grafting according to a previous study with slight modifications [38]. First, precursor chitosan (0.5 g) was dissolved in 2 wt % acetic acid (50 mL) at 70 °C for 24 h with magnetic stirring, the solution was cooled to room temperature (RT), and a mixture of ascorbic acid (AA) (54 mg), water (0.9 mL), and 25% H_2_O_2_ (0.1 mL) was added to yield chitosan (CS). After 30 min, an aqueous solution (20 mL) of phenolic compound (0.35 mmol) was added dropwise, and the reaction vessel was wrapped with aluminum foil to protect the reactants from light. The mixture was magnetically stirred at RT for 24 h and dialyzed against water, which was changed at least eight times, for 48 h using a membrane with a molecular weight cut-off of 14 kDa (Roth; Karlsruhe, Germany). The resulting polymer was filtered through a 0.22 μm diameter syringe filter and freeze-dried. Depending on the phenolic compound that was used, the polymers were denoted as CS-H (hydroquinone-modified chitosan), CS-G (gallic acid-modified chitosan), and CS-P (phloroglucinol-modified chitosan).

### Synthesis of magnetic nanoparticles

Iron oxide particles were prepared by a co-precipitation method with slight modifications [39]. Briefly, FeCl_2_·4H_2_O (1.191 g) and FeCl_3_·6H_2_O (3.242 g) were dissolved in water (175 mL), the solution was heated to 70 °C for 10 min, and 25% NH_4_OH (10 mL) in water (25 mL) was added dropwise. The mixture was heated at 90 °C for 1 h, and the particles were washed with water four times (50 mL for each wash) and dispersed in water (200 mL). To oxidize magnetite, 37% hydrochloric acid (150 µL) in water (5 mL) and 30% hydrogen peroxide (1.5 mL) were added. The mixture was heated at 90 °C for 1 h, and the resulting maghemite (γ-Fe_2_O_3_) nanoparticles were washed with water two times (100 mL for each wash) and dispersed in water (50 mL) to a concentration of 26.5 mg of γ-Fe_2_O_3_ per milliliter of water.

### Surface modification of magnetic nanoparticles

**Figure 8:**
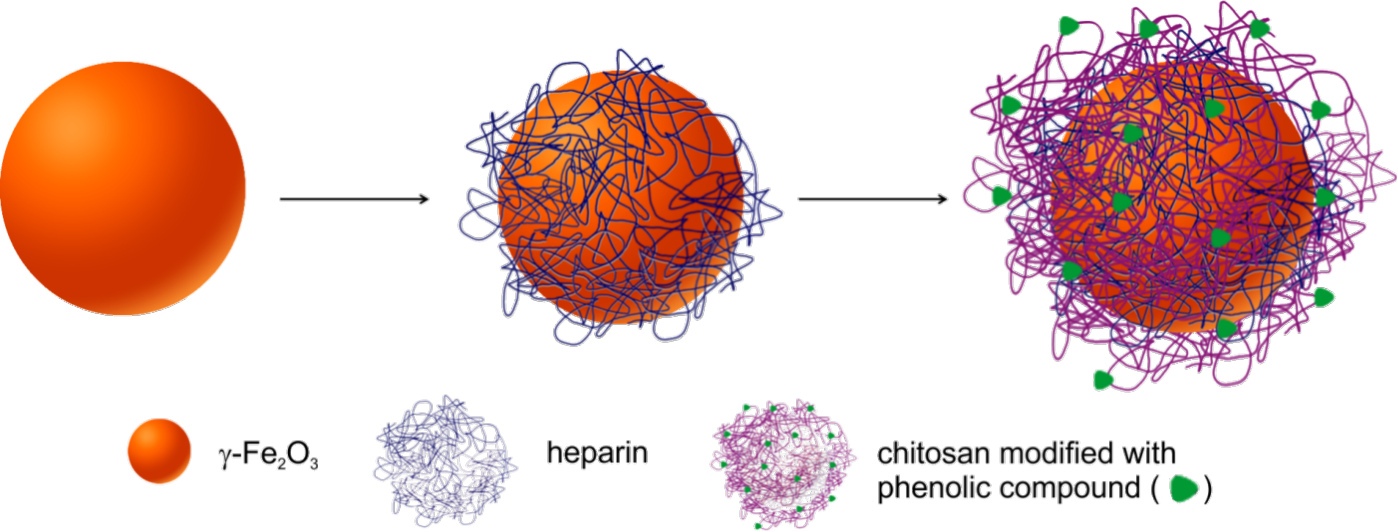
A schematic of the iron oxide nanoparticle coating with heparin and gallic acid-modified chitosan.

Thin polymer layers of opposite charges were sequentially deposited on the magnetic nanoparticles by the LbL adsorption technique (Figure 8). The aqueous γ-Fe_2_O_3_ (20 mg) dispersion was diluted with water to 4 mL and sonicated with a W-385 sonicator (Heat Systems-Ultrasonics; Farmingdale, NY, USA; amplitude 10%) for 5 min. Heparin salt (4.05 mg) was dissolved in water (5 mL), added to the magnetic particles after sonication, and the mixture was vigorously vortexed for 2 h. The resulting γ-Fe_2_O_3_@Hep nanoparticles were washed with water two times (25 and 10 mL), redispersed in water (10 mL), and divided into two parts that were magnetically separated. The supernatant was decanted, and the particles were redispersed in water (5 mL) with sonication for 5 min. The concentration of the γ-Fe_2_O_3_@Hep particles in water was 2 mg/mL.

The second layer, consisting of a phenolic compound-modified chitosan, was introduced immediately after the first coating with heparin. An aqueous solution (5 mL) of modified chitosan (8.1 mg) was magnetically stirred at 50 °C for 20 min and cooled to RT, while the γ-Fe_2_O_3_@Hep nanoparticle dispersion was sonicated for 5 min. After 2 min of sonication, the chitosan solution was added, and the mixture was vigorously vortexed for 1 h. The resulting nanoparticles were washed with water three times (10 mL for each wash) and redispersed in water with sonication for 5 min. Depending on the type of chitosan (CS-G, CS-H, and CS-P) used for the modification, the nanoparticles were denoted as γ-Fe_2_O_3_@Hep-CS-G, γ-Fe_2_O_3_@Hep-CS-H, and γ-Fe_2_O_3_@Hep-CS-P, respectively. The typical particle concentration was 4.4 mg/mL.

### Characterization methods

**Attenuated total reflection Fourier transform infrared spectroscopy (ATR-FTIR) and proton nuclear magnetic resonance spectroscopy (^1^H NMR).** ATR-FTIR spectra were measured using a PerkinElmer Paragon 1000 PC spectrometer (Waltham, MA, USA) with a resolution of 4 cm^−1^, 32 or 96 accumulations for chitosans or nanoparticles, respectively, strong apodization, and a DTGS detector. ^1^H NMR spectra of the polymers were acquired using a Bruker Avance III 600 spectrometer (Billerica, MA, USA) with a 90° width, 10 μs pulse, 10 s relaxation delay, 2.18 s acquisition time, and 100 scans. DCl (2 wt %) in D_2_O was used as a solvent. The chemical shift was calibrated from the 4,4-dimethyl-4-silapentane-1-sulfonic acid signal at δ = 0 ppm.

**Size-exclusion chromatography (SEC).** The molecular weight of chitosan before and after free-radical grafting was measured using a chromatographic system consisting of a DeltaChrom pump (Watrex; Prague, Czech Republic), a MIDAS autosampler (Spark Holland; Emmen, Netherlands), a PL Aquagel-OH MIXED-H column (Ercatech; Bern, Switzerland) with 8 μm particles and a separation range of 0.4–10,000 kDa, a DAWN HELEOS II light-scattering detector and an Optilab T-rEX detector (Wyatt Technology; Goleta, CA, USA). Water was the mobile phase, and the injection-loop volume was 0.1 mL. The data were processed using Astra 6.1 software (Wyatt Technology).

**The antioxidant properties of phenolic compound-modified chitosans** were examined by two assays. The first assay was based on a Folin–Ciocalteu (FC) reagent that enables the determination of the total phenolic content, and the second assay analyzed the ability to scavenge the radical form of DPPH. The FC test was performed only for the polymer, and the DPPH assay was performed for chitosans and nanoparticles.

For the FC assay, chitosan (ca. 10 mg) was dissolved in 2% acetic acid (2 mL) at 50 °C for 30 min, and the solution was cooled to RT. An aliquot of the solution (20 μL) was transferred to a 2 mL Eppendorf tube containing water (1.58 mL) and FC reagent (100 μL). Then, 17 wt % sodium carbonate solution (300 μL) was added, and the mixture was vortexed for 140 s and kept in the dark for 1.5 h. Absorbance was measured using a Specord 250Plus UV spectrometer (Analytik Jena AG; Jena, Germany) at a wavelength of 765 nm against a control solution (without chitosan). A series of gallic acid solutions of different concentrations (0.0–0.5 mg/mL) was used to obtain the calibration curve. The results were expressed as the gallic acid equivalent (GAE). Each experiment was performed in triplicate.

For the DPPH assay, 0.1 mM DPPH solution in ethanol was left to stand for 2 h. Chitosan (ca. 20 mg) was dissolved in 2% acetic acid (2 mL), and a series of solutions was prepared with a dilution factor of 2. Ethanol (1.2 mL) and DPPH solution (0.6 mL) were added to a 2 mL Eppendorf tube containing chitosan solution (0.2 mL), and the mixture was vigorously vortexed for 1 min and stored in the dark for 30 min. The absorbance was measured at a wavelength of 517 nm against ethanol using the same spectrometer as above. A mixture of 0.1 mM DPPH (0.6 mL and ethanol (1.4 mL) served as a control. DPPH inhibition was determined as follows:

[1] $$\text{inhibition}=\frac{A_{0}-A_{1}}{A_{0}}\cdot 100\%,$$

where *A*_0_ and *A*_1_ are the absorbance of the control and the sample, respectively. The results from the DPPH assay were expressed as the half maximal inhibitory concentration (IC_50_), which is the amount of substance required to decrease the amount of DPPH radicals by half. IC_50_ values were calculated from the linear dependence of the absorbance on the concentration of a phenolic compound. Each experiment was performed in triplicate.

**Transmission electron microscopy (TEM).** An FEI Tecnai G2 Spirit TEM microscope (Brno, Czech Republic) was used to analyze the morphology of the particles. Number-average (*D*_n_) and weight-average diameter (*D*_w_) and dispersity (*Ð* = *D*_w_/*D*_n_) were calculated from at least 500 particles using Atlas software (Tescan; Brno, Czech Republic), as described previously [40].

**Dynamic light scattering (DLS).** Hydrodynamic diameter (*D*_h_), polydispersity (*PD*), and ζ-potential of the nanoparticles in water were measured using a Zetasizer-NS apparatus (Malvern Instruments; Malvern, UK). Before the measurement, the particle dispersion was sonicated for 4 min and left to stand for 10 min. The results are expressed as arithmetic means of three independent measurements.

**Thermogravimetric analysis (TGA)** was conducted in air flow at a heating rate of 10 °C/min using a PerkinElmer TGA 7 thermogravimetric analyzer (Waltham, MA, USA) operated with Pyris 1 software.

**Magnetic properties.** Magnetization as a function of temperature (10–260 K) and magnetic field (up to 5.5 T) was obtained for colloids frozen in quartz tubes using an MPMS superconducting quantum interference device (SQUID; Quantum Design; San Diego, CA, USA). The temperature dependence was analyzed using zero-field-cooled (ZF-C) and field-cooled (F-C) measurements under an applied magnetic field of 5.0 mT.

**The antioxidant properties of phenolic compound-modified particles** were determined using the DPPH assay as described above, except that a magnetic colloid (0.2 mL) was used instead of chitosan. The mixtures were vortexed for 20 min, and the particles were magnetically separated.

**Particle uptake by cultured cells.** L-929 (human fibroblast cells) and LN-229 (human glioma cells) cells were supplied by the Food Industry Research and Development Institute (Hsinchu, Taiwan). L-929 cells were cultured in minimum essential medium Eagle supplemented with 10% equine serum, 1 mM sodium pyruvate and 1% antibiotic/antimycotic solution, whereas LN-929 cells were cultured in Dulbecco’s modified Eagle’s medium supplemented with 10% FBS and 1% penicillin/streptomycin/amphotericin B. Both L-929 and LN-229 cells were cultured at 37 °C in an incubator under a 5% CO_2_ atmosphere and subcultured every three to four days. To determine cell-associated magnetic nanoparticles (MNP_cell_) as an indicator of cellular uptake of the particles, cells were cultured in a 24-well culture plate until reaching 80–90% confluence and incubated with γ-Fe_2_O_3_, γ-Fe_2_O_3_@Hep, γ-Fe_2_O_3_@Hep-CS-G, γ-Fe_2_O_3_@Hep-CS-H, and γ-Fe_2_O_3_@Hep-CS-P particles (100 μg/mL) for 3 h in the absence or presence of an NdFeB magnet (ca. 3.4 kG) placed underneath the cells [41]. A magnetic field was applied for 5 min after the addition of nanoparticles to facilitate particle sedimentation. The cells were then harvested and treated with 10% HCl at 55 °C for 4 h, which was followed by the addition of ammonium persulfate (1 mg/mL) and 1 M potassium thiocyanate solution. The amount of cell-associated iron was determined with a VICTOR3 Multilabel plate reader (PerkinElmer; Waltham, MA, USA) at a wavelength of 490 nm. A calibration curve was prepared under identical conditions.

**Cytotoxicity** was determined by a CCK-8 assay (Sigma-Aldrich) according to the manufacturer’s instructions. Briefly, cells were cultured in a 24-well plate to 80–90% confluence and incubated with nanoparticles (100 μg/mL) for 3 h in the absence or presence of the NdFeB magnet. The cells were then washed with PBS and incubated with CCK-8 solution-containing medium (10%) for an additional hour. The optical density (OD) of each sample was determined with a VICTOR3 Multilabel plate reader at a wavelength of 450 nm. The percentage of cell viability was calculated as follows: (OD of supernatant from cells incubated with particles/OD of supernatant from cells without incubation with particles) × 100.

**Analysis of antioxidant properties by flow cytometry.** To determine the cellular ROS scavenging activity, L-929 and LN-229 cells were incubated with γ-Fe_2_O_3_, γ-Fe_2_O_3_@Hep, γ-Fe_2_O_3_@Hep-CS-G, γ-Fe_2_O_3_@Hep-CS-H, and γ-Fe_2_O_3_@Hep-CS-P particles (100 μg/mL) for 3 h. The cells were then washed with PBS twice and incubated with 2 mM H_2_O_2_ for 30 min. After removal of H_2_O_2_-containing medium, the cells were incubated with 5 μM CM-H_2_DCFDA for 1 h, trypsinized, and analyzed by an Attune™ NxT Acoustic Focusing Cytometer (Thermo Fisher Scientific; Waltham, MA, USA). The side scatter (SSC-A) and forward scatter (FSC-A) indicated cellular complexity and cell volume, respectively. Absorbance was detected at a wavelength of 535 nm for the determination of the intracellular ROS signal (DCF-A), which was gated at 400 and 1200 for L-929 and LN-229 cells, respectively.
